# Choosing wisely in Allergology: a Slow Medicine approach to the discipline promoted by the Italian Society of Allergy, Asthma and Clinical Immunology (SIAAIC)

**DOI:** 10.1186/s12948-015-0034-8

**Published:** 2015-11-20

**Authors:** Enrico Heffler, Massimo Landi, Silvana Quadrino, Cristoforo Incorvaia, Stefano Pizzimenti, Sandra Vernero, Nunzio Crimi, Giovanni Rolla, Giorgio Walter Canonica

**Affiliations:** Department of Clinical and Experimental Medicine, Respiratory Medicine and Allergy, University of Catania, Catania, Italy; Pediatrician Primary Care, Turin, Italy; “Change” Institute, Turin, Italy; Allergy/Pulmonary Rehabilitation, ICP Hospital, Milan, Italy; ASL-TO3, Allergy Outpatients’ Clinic, “Edoardo Agnelli” Hospital, Pinerolo, TO Italy; “Slow Medicine Italy”, Turin, Italy; Department of Medical Sciences, Allergy and Clinical Immunology, University of Torino, Turin, Italy; Allergy and Respiratory Diseases Clinic, DIMI-Department of Internal Medicine, IRCCS AOU S.Martino-IST, Genoa, Italy

**Keywords:** Allergy, Slow medicine, Chossing wisely, Appropriateness, Asthma, Food allergy

## Abstract

**Background:**

One of the main problem health care systems are facis is the mis-use and over-use of medical resources (including useless exams, surgical interventions, medical treatments, screening procedures…) which may lead to high health care related costs without increased patients’ benefit and possible harm to the patients themselves. The “Choosing wisely” campaign, in Italy denominated “Doing more does not mean doing better”, tries to educate doctors and citizens at a correct use of medical resources.

**Methods:**

the Italian Society of Allergy, Asthma and Clinical Immunology (SIAAIC) adhered to the “Doing more does not mean doing better” campaing and made a list of the 5 allergological procedures with the highest evidence of inappropriateness.

**Results:**

the 5 recommendations were: “Do not perform allergy tests for drugs (including anhestetics) and/or foods when there are neither clinical history nor symptoms suggestive of hypersensitivity reactions”; “Do not perform the so-called “food intolerance tests” (apart from those which are validated for suspect celiac disease or lactose enzymatic intolerance)”; “Do not perform serological allergy tests (i.e.: total IgE, specific IgE, ISAC) as first-line tests or as “screening” assays”; “Do not treat patients sensitized to allergens or aptens if there is not a clear correlation between exposure to that specific allergen/apten and symptoms suggestive of allergic reaction”; “Do not diagnose asthma without having performed lung function tests”.

**Conclusions:**

An important role scientific societies should play is to advise on correct diagnostic and therapeutical pathways. For this reason SIAAIC decided to adhere to the Slow Medicine Italy campaign “Doing more does not mean doing better” with the aim of warning the scientific community and the citizens/patients about some allergological procedures, which, when performed in the wrong clinical setting, may be not only useless, but unnecessarily expensive and even harmful for patients’ health.

## Background

The concept of “Slow Medicine” has been coined by Dr. Alberto Dolara, an Italian cardiologist that in 2002 invited his colleagues to give the deserved value to the time spent in improving the patient-doctor relationship, implementing a more “human and thoughtful medicine” [1], but these underlying ideas were somehow anticipated from some phylosophers such as Ivan Illich that, with his “Medical nemesis” published in 1974, argued that the medicalization in recent decades of so many of life’s vicissitudes—including birth and death—and the so called “hubris of medicine” frequently caused more harm than good and rendered many people in effect lifelong patients [2].

In the last few years, we assisted to a worldwide dramatic increase in interest in the “Slow Medicine” concept, and this was also endorsed by the former British Medical Journal (BMJ) editor in chief, professor Richard Smith, which wrote: “slow medicine—like slow food and slow lovemaking—is the best kind of medicine for the 21st century” [3].

One of the main problem the “Slow Medicine” approach is trying to face to promote possible solutions, is the mis-use and over-use of medical resources (including useless exams, surgical interventions, medical treatments, screening procedures…) which is well known to lead to both high health care related costs without increased patients’ benefit [4] and possible harm to the patients themselves [5, 6].

Into this context, the “Choosing wisely” campaign started in the USA in 2012 [7, 8] and then spread in several other countries, including Italy with the name “Doing more does not mean doing better” (“Fare di più non significa fare meglio” in Italian) [9–11], has the main goal of identifying the most probable inappropriate medical procedures for each specialty, protecting patients’ interests through a partnership between health professionals and patients and users [12].

In order to create an accurate list of the five medical procedures with the highest probability of inappropriateness for each specialty, Slow Medicine Italy [13] invited the most relevant Italian scientific societies to adhere to the “Doing more does not mean doing better” campaign. The Italian Society of Allergy, Asthma and Clinical Immunology (SIAAIC), the largest Italian scientific society in the field with more than 700 active members, enthusiastically adhered to the campaign. In this article we will described and discuss the methodology used and the obtained results to make the list of five allergological procedures with the highest probability of inappropriateness.

## Methods

After formal adhesion of SIAAIC to the “Doing more does not mean doing better” campaign, a working group of senior and junior members of the Society and experts in the field of Allergology has been established. The board discussed and identified a first list of allergological procedures with a possible high degree of inappropriateness. The board members performed an extensive search on PubMed and Cochrane Database, without any limit of age, gender or time of publication, in order to find enough evidence of inappropriateness for each identified allergological procedure (search keywords were depending on the subjects of the identified allergologica procedures; all types of articles were included into the evaluation), selecting the five with the highest evidence of inappropriateness taking also in consideration the frequency and the social impact of each of them, and reporting them as a “do not” suggestion. The list of the identified 5 most inappropriate allergological procedures is reported in Table 1.

**Table 1:** The list of identified 5 most inappropriate allergological procedures

Do not perform allergy tests for drugs (including anhestetics) and/or foods when there are neither clinical history nor symptoms suggestive of hypersensitivity reactions
Do not perform the so-called “food intolerance tests” (apart from those which are validated for suspect celiac disease or lactose enzymatic intolerance)
Do not perform serological allergy tests (i.e.: total IgE, specific IgE, component-resolved diagnosis) as first-line tests or as “screening” of inhalant & food immediate hypersensitivity assays
Do not treat patients sensitized to allergens or aptens if there is not a clear correlation between exposure to that specific allergen/apten and symptoms suggestive of allergic reaction. This recommendation is particularly strong for allergen immunotherapy and elimination diets
Do not diagnose asthma without having performed lung function tests (including bronchodilating test and/or bronchial challenge)

This list has been approved by both the Executive Committees of SIAAIC and Slow Medicine Italy, published on their websites [14, 15] and spread as a poster sent to all SIAAIC members.

## Results and discussion

We here briefly discuss each of the identified 5 most inappropriate allergological procedures.

### Do not perform allergy tests for drugs (including anhestetics) And/or foods when there are neither clinical history nor symptoms suggestive of hypersensitivity reactions

In absence of clinical history or symptoms suggestive of hypersensitivity reactions (i.e.: urticaria, angioedema, other typical muco-cutaneous manifestations, hypotension, respiratory symptoms, contemporary involvement of two or more organs, or any other consisting organ damage) allergometric tests do not have any clinical value and have a poor predictive value of future allergic reactions [16]. In this context, a positive allergometric test indicates only an immunological sensitization to the tested antigen.

On the other hand, a negative test is indicative only of the current absence of sensitization but it does not exclude the possibility of future allergic reactions.

The harms connected to this procedure are:

1. Non adequate therapeutical approaches (including diets [16]) which are potentially harmful because they may preclude the use of drugs or the assumption of foods the patient is not allergic to;
2. it has been described, in two small non randomized controlled trials and therefore with low quality of evidence, the possibility of new sensitizations to the tested antigens induced by the tests themselves [17, 18].

### Do not perform the so-called “food intolerance tests” (apart from those which are validated for suspect celiac disease and lactose enzymatic intolerance)

Several assays and techniques are constantly proposed to many patients to identify supposed food intolerance. These methods include, for example, VEGA-test, Cytotoxic test, serum specific IgG4 dosage, chemical analysis of hair, applied kinesiology, iridology, and “bioresonance” analysis. None of these methods reached sufficient evidence of efficacy, accuracy and repeatability in diagnosis food allergy/intolerance [19–28].

The use of these methods, giving unreliable and not clinically relevant results, put the patient at risk of inappropriate diets which are potentially harmful for health, without finding a solution to the symptoms reported by the patient [29, 30].

This recommendation is particularly important in a context of non adequate perception and knowledge of food allergy/intolerance symptoms by both patients and general practitioners [31].

### Do not perform serological allergy tests (i.e.: total ige, specific ige, component-resolved diagnosis) as first-line tests or as “screening” of inhalant and food immediate hypersensitivity assays

Cutaneous allergometric tests, if possible, should be considered as the first diagnostic tests in case of consisting clinical history and symptoms with a suspect allergic reaction, as they give faster results, they are less invasive and cheaper than serological tests. Moreover, there is a moderate evidence that, at least for food allergies, that skin tests have at least the same diagnostic accuracy of serological tests [32, 33].

Exceptions to this recommendations are:

1. Situations in which cutaneous tests are not feasible, such as hypo- or hyper-reactive cutaneous states (i.e.: chronic assumption of antihistamines or systemic corticosteroids, or the presence of frank dermographism);
2. Non availability of any accurate extracts to perform skin tests against the availability of serological tests for the same allergen [16, 34].
3. When the clinical history suggests an unusually greater risk of anaphylaxis from skin testing [35].

Total IgE assessment is of limited clinical utility in most of the cases, as it is not necessarily indicative of allergic sensitization: allergic patients may have both normal or elevated total IgE levels, and patients with high total IgE levels are not necessarily atopic subjects [16, 34, 36].

Measuring total IgE is otherwise indicated for the diagnosis of allergic bronchopulmonary aspergillosis, hyper IgE syndrome, as well for verification that the patient with severe allergic asthma is a suitable candidate for anti-IgE therapy with total serum IgE levels between 30 and 1500 IU/ml.

Moreover, all serum allergological tests should be interpreted by specialists/experts in Allergy and Clinical Immunology as a wrong interpretation can lead a non expert doctor to offer therapeutical and/or dietetical inappropriate approaches which may be harmful for the patient’s health.

### Do not treat patients sensitized to allergens or aptens if there is not a clear correlation between exposure to that specific allergen/apten and symptoms suggestive of allergic reaction. This recommendation is particularly strong for allergen immunotherapy and elimination diets

The finding of a positive allergometric test for an allergen or apten whose exposure is not associated with symptoms compatible with allergic reaction is only indicative of immunological sensitization and not necessarily of clinical manifestations related to hypersensitivity reaction [16, 34]. Therefore, there is no indication to treat these patients.

Moreover, as far as food allergy, given the limitation of cutaneous and serological tests, oral food challenges (ideally Double-Blind Placebo-Controlled Food-Challenges) are still the gold standard in IgE and non IgE mediated food allergy in order to establish a firm diagnosis, determine threshold reactivity, assess tolerance and the response to immuno-modulation [37].

Suggesting a treatment (including immunotherapic or dietetic strategies) to these patients may expose them to the risk of useless and potentially harmful therapies [38–40].

In particular, elimination diets, when they are not indicated, may expose the patient to nutritional deficiencies with no improvement of symptoms for which the allergometric investigations had been carried out [39, 40].

### Do not diagnose asthma without having performed lung function tests (including bronchodilating test and/or bronchial challenge)

To rely only upon asthma-like symptoms (i.e.: dyspnea, chest tightness, cough, wheezing) is not sufficient to make a correct diagnosis of asthma, as these symptoms may be from alternate causes, such as chronic obstructive pulmonary disease (COPD), congestive heart failure, extrathoracic airway hyperresponsiveness syndromes (e.g. vocal cord dysfunction, VCD), gastroesophageal reflux disease, hyperventilation syndrome etc. [41–44]. This behavior can be harmful for patients as they may be receive a wrong treatment for their complaints; this is particularly important when patients are affected by other relevant comorbidities as it happens in elderly [45].

International asthma guidelines stress the need of performing complete lung function assessment to identify bronchial hyperreactivity and/or reversibility of bronchial obstruction [46]. Patients with asthma-like symptoms and normal spirometry should underwent to an aspecific bronchial challenge (i.e.: with methacholine) while those with an obstructive spirometric pattern should be evaluated for the degree of reversibility during a bronchodilating test (i.e.: with salbutamol). Beyond the increased costs of care, the consequences of misdiagnosing asthma include delaying a correct diagnosis and treatment [43].

## Conclusions

In this article we described the methodology used and the obtained results to make the list of five allergological procedures with the highest probability of inappropriateness [14, 15], in the context of the “Doing more does not mean doing better” campaign proposed by Slow Medicine Italy [9–11]. The five selected procedures were identified by an expert panel of senior and junior Italian allergologists.

The modern medicine is imbued with inappropriate medical procedures, wastes, conflicts of interest and fraud deriving from the economic and financial interactions between prescribers, purchasers of health technologies and the industry [13]. Another important cause which may induce the doctors to order inappropriate procedures lies in malpractice claims from patients (defensive medicine). This behavior is often encouraged by the message, which comes from the “media” and easily received by patients, that in medicine “more is always better” and that “doing less is always an index of medical malpractice” [47].

An important role scientific societies should play is to produce and disseminate Diagnostic-Therapeutic-Healthcare Protocols/Pathways, based on the best evidence based scientific knowledge, to guarantee the patients receive the correct diagnosis and the appropriate treatment. For this reason SIAAIC decided to adhere to the Slow Medicine Italy campaign “Doing more does not mean doing better” with the aim of warning the scientific community and the citizens/patients about some allergological procedures, which, when performed in the wrong clinical setting, may be not only useless, but unnecessarily expensive and even harmful for patients’ health.

We think that doctors and patients should take more time to define together the most appropriate pathway which lead to the right diagnosis and to the most appropriate treatment. Doctors should always explain the patients what the exams they order mean in case they turned normal or altered and why some exams are not useful at all for the specific diagnosis the patients are looking for. An open and trustful patient-physician relationship appears to be important to avoid useless exams and to achieve more accurate diagnoses .

## Abbreviations

SIAAIC: Italian Society of Allergy, Asthma and Clinical Immunology
COPD: chronic obstructive pulmonary disease
VCD: vocal cord dysfunction
